# Neurologic sequelae of phosphide poisoning: A case report

**DOI:** 10.3389/fneur.2022.888493

**Published:** 2022-09-16

**Authors:** Sahel Shafiee Dolat Abadi, Nasim Zamani, Sahar Abbasi, Maziar Shojaei, Hossein Hassanian-Moghaddam

**Affiliations:** School of Medicine, Shahid Beheshti University of Medical Sciences, Tehran, Iran

**Keywords:** aluminum phosphide, poisoning, brain sequelae, encephalopathy, brain death

## Abstract

**Background:**

Aluminum phosphide (ALP) is extremely toxic with a high mortality rate, mainly due to its cardiovascular complications. Some neurologic effects have also been reported with this pesticide.

**Case presentation:**

We present a 23-year-old male who presented with confusion after ingestion of a toxic dose of ALP. Computerized tomography scan demonstrated diffuse bilateral hypoattenuation of the cerebellar hemispheres, midbrain, thalamus, and globus pallidus resulting in tonsillar and transtentorial herniation and eventually brain death four days after admission.

**Conclusions:**

This is the first documented case of neurologic sequela following phosphide poisoning that emphasizes the importance of brain imaging studies for patients with loss of consciousness.

## Background

Aluminum phosphide (ALP) tablet, commonly known as “rice tablet”, is widely available in 3 gr greenish-gray tablets with garlic odor. The initial manifestations of acute aluminum phosphide poisoning appear in 10–15 min and are mainly cardiovascular and respiratory in nature, although multi-organ failure may also develop (1). ALP can be diagnosed in the emergency department using a “Silver nitrate test,” which changes the color of silver nitrate to dark brown. Furthermore, carboxyhemoglobin level may be falsely elevated due to the formation of dyshemoglobinemia. Lactate level can be used to determine the severity of poisoning. Methemiglobinemia may be seen in G6PD deficient patients. ECMO has shown a promising intervention to treat patients with refractory hypotension (2–4). The most common causes of death are cardiovascular collapse, refractory shock, severe acidemia, and adult respiratory distress syndrome (ARDS) (5). Cough, dyspnea, cyanosis, pulmonary edema, respiratory failure, and ARDS are some of the respiratory features that might be seen (6). Neurologic complications have rarely been reported with this toxicity.

## Case presentation

A 23-year-old male was admitted with drowsiness, confusion, and nausea/vomiting an hour after suicidal ingestion of one ALP tablet. He had a history of untreated major depressive disorder (MDD) and no family history of other diseases. On admission, the blood pressure (BP), pulse rate (PR), O_2_ saturation, respiratory rate (RR), temperature, and blood sugar (BS) levels were 92/57 mmHg, 91 bpm, 92%, 18/min, 36.7°C, and 99 mg/dL, respectively. He was intubated and a central venous catheter as well as a nasogastric tube were inserted. He was treated by administration of fluids (1 L normal saline), activated charcoal (0.5 gr/kg), potassium permanganate, sodium bicarbonate (2 mEq/kg), intravenous ondansetron, pantoprazole, calcium gluconate (2 gr q 8h), and magnesium sulfate (2 gr q 12h). The 12-lead electrocardiogram was normal. An initial venous blood gas (VBG) analysis showed respiratory alkalosis which changed to severe metabolic acidosis within 8 h (pH = 7.18, pCO_2_ = 28.7, HCO_3_ = 12.5, BE = 16.1). Renal function was mildly reduced (creatinine was 1.4 mg/dL and GFR was 67 ml/min/1.73). His urine toxicology screen was negative for benzodiazepines, opiates, amphetamine, methamphetamine, cannabis, and methadone. Table 1 displays the on-arrival lab exams.

**Table 1:** On-arrival lab test results.

**Lab test**	**Result (normal range)**
White blood cells ( ×1,000/mm^3^)	10.7 (4.5–11)
Hemoglobin (g/dL)	14.2 (13.5–17.5)
Hematocrit (percent)	41.3 (45–52)
Platelet count ( ×1,000/mm^3^)	232 (150–450)
Urea (mg/dL)	26 (7–20)
Creatinine (mg/dL)	1.4 (0.9–1.3)
Aspartate transaminase (IU)	11 (10–40)
Alanine transaminase (IU)	10 (10–40)
Lactate dehydrogenase (U/L)	290 (<480)
Creatine phosphokinase (U/L)	94 (25–195)
Alkaline phosphatase (IU/L)	117 (20–140)
Total bilirubin (mg/dL)	1 (0.1–1.2)
Direct bilirubin (mg/dL)	0.4 (<0.3)
Serum sodium (mEq/L)	140 (135–145)
Serum potassium (mEq/L)	3.7 (3.5–5)
Prothrombin time (seconds)	11.4 (11–13.5)
International normalized ratio	1.06 (0.8–1.1)
Partial thromboplastin time (seconds)	29.6 (25–35)
Reticulocytes	0.9 (0.5–1.5)
Coombs direct	negative
Coombs Indirect	negative
Venous PH	7.44 (7.32–7.43)
Venous pCO_2_ (mmHg)	23 (41–50)
Venous pO_2_ (mmHg)	28 (20-40)
Venous HCO_3_ (mEq/L)	22 (23–27)

A brain CT was requested due to confusion; however, it was not done because of the patient's unpredictable condition.

A hypotensive state occurred 3 h after the primary hypotension was managed, mandating increasing administration of norepinephrine infusion (5–15 mic/kg/min) and transfer to the ICU. He developed an atrial fibrillation (AF) rhythm 2 h after being admitted to the ICU and was given amiodarone (150 mg as a starting dose followed by 1 mg/min for 6 h and 0.5 mg/min for 18 h). An echocardiogram was performed 8 h after admission which revealed an ejection fraction of 20% and global hypokinesia which was started to be treated by glucose-insulin-potassium protocol (1–5 unit/kg insulin and 0.5–1 gr/kg glucose) and frequent 1-h checks of vitals and blood glucose (7, 8).

**Figure 1:**
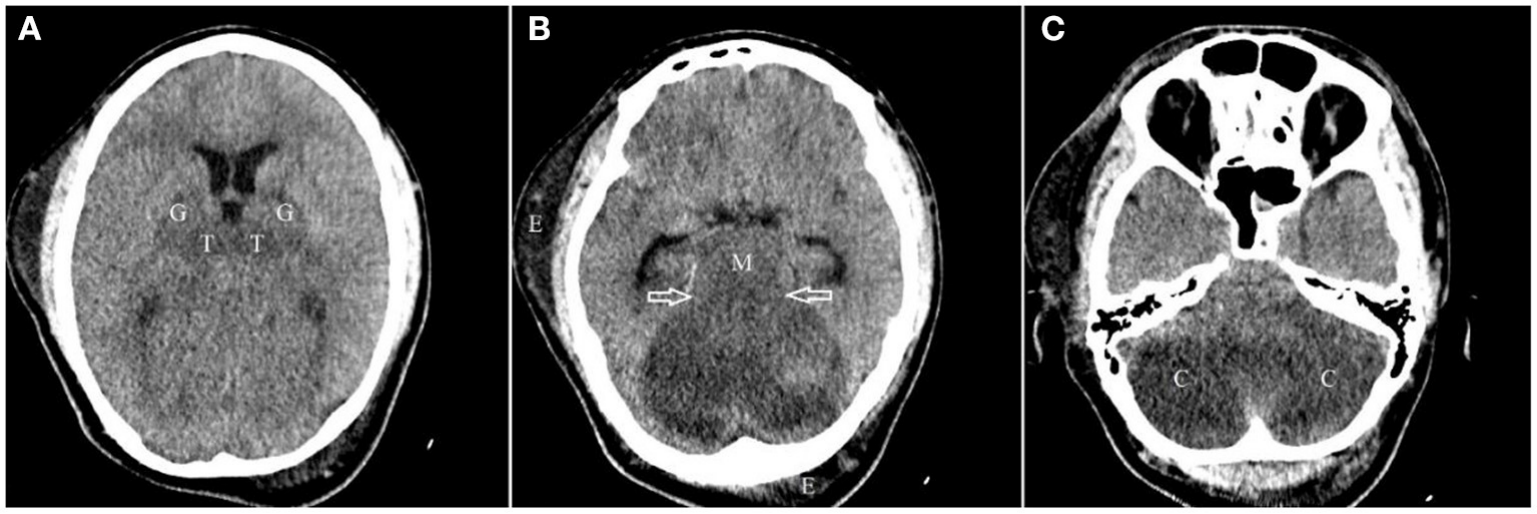
Axial images of non-contrast brain CT scan from top to bottom **(A–C)** demonstrate near symmetric hypodensity involving both thalami (T) and globi pallidi (G), bilaterally, midbrain (M) and both cerebellar hemispheres (C) with evidence of ascending transtentorial herniation [hallow arrows in **(B)**]. Subcutaneous edema (E) in right temporal and left occipital regions are also seen.

The lowest recorded blood sugar level and blood pressure during his ICU stay were 66 mg/dL and 91/48 mmHg on the second and third day of hospitalization which were promptly treated. He was admitted to the ICU with GCS of 6/15 which improved to 9/15 by the end of the first day. On day two, his GCS decreased to 5/15 and he developed anisocoria. On the following day, the GCS dropped to 3/15. His pupils were mydriatic and non-reactive to light. An emergency CT on the third day showed diffuse bilateral hypoattenuation in the cerebellar hemispheres, midbrain, thalamus, and globus pallidus nuclei, suggesting ischemic or toxic insult. Tonsillar and transtentorial herniations were also detected in association with mild obstructive hydrocephalus (Figure 1).

EEG study identified severe diffused encephalopathy of non-specific etiology. On the fourth day, the patient experienced bradycardia and was once successfully resuscitated. Later, he was pronounced brain-dead and a potential candidate for organ donation. Seventeen hours later, he re-developed bradycardia and asystole which did not respond to resuscitation and the patient was pronounced dead by the end of the fourth day.

## Discussion and conclusion

Phosphine seems to mainly inhibit cytochrome oxidase and alter hemoglobin's heme portion. It also leads to oxidative stress and an increase in extra-mitochondrial free oxygen radicals, causing lipid peroxidation in cell membranes and protein denaturation. These mechanisms impair adenosine triphosphate (ATP) synthesis and mitochondrial function, ultimately leading to DNA and cell membrane damage and cell death (9).

Mitochondria are essential for energy metabolism and oxidative phosphorylation. The nervous system contains metabolically active cells, that are enormously reliant on ATP produced by mitochondrial oxidative phosphorylation (10). Mitochondrial disorders usually manifest with neurological symptoms including external ophthalmoplegia, optic atrophy, seizures, fluctuating encephalopathy, stroke-like episodes, ataxia, and spasticity (11).

In ALP-poisoned patients with hypoxia or hypotension, neurological symptoms may include headache, drowsiness, dizziness, paresthesia, tremor, and weakness and only noticeable in patients with hypoxia or hypotension (12). There are few reports of long-term neurological abnormalities in ALP-poisoned patients. In 2014, Abedini et al. reported a case of ischemic stroke in the right middle cerebral territory caused by an ALP poisoning 11 days earlier (13). Brautbar and Howard (14) reported survivors of phosphine poisoning with persistent severe headaches and peripheral neuropathy in the long-term. Neuropathological studies revealed neuronal degeneration, absence of Nissl granules and processes, and the degenerated nucleus eccentric location. These changes may be caused by phosphine which impairs cellular oxygen usage resulting in neuro-cellular dysfunction (15). This is while, to date, no cases of primary neurologic involvement (confusion or low GCS) have been reported with near normal respiratory and cardiac function. Our patient was referred with confusion. He had only taken one 3 gr ALP tablet with no other medications or substances. He developed hypodensities in the posterior fossa, bilateral thalami, and globus pallidus nuclei that likely represent ischemic or toxic cellular injury or death even though no MR-imaging or neuropathological correlation was available for confirmation. On the other hand, hypoxic-ischemic infarctions do not typically occur in these areas and considering that his blood pressure and sugar were tightly controlled and were never persistently low. There is broader concentration of mitochondria, vascular supply, neurotransmitters, and chemical content in the putamen and globus pallidus. Because of their high metabolic activity and increased glucose and oxygen intake, they are sensitive to metabolic abnormalities and a variety of systemic diseases. Patients with poorly controlled diabetes, acute chorea, and altered GCS may sometimes show bilateral or unilateral pallidal or caudate hyper-attenuation on their CT scan (16). Extreme hypoglycemia is characterized by bilateral T2 prolongation in the cerebral cortex, hippocampi, and basal ganglia on MR imaging (17). In adults, hypoxic-ischemic encephalopathy (HIE) can be triggered by circulatory or respiratory failure caused by cardiac arrest, drowning, or asphyxia. The watershed areas can be affected by mild HIE. Gray matter structures, such as the cerebral cortex, basal ganglia, and hippocampi are typically affected by severe HIE (18).

Interestingly, zinc phosphide, the other formulation of metal phosphides may have other imaging findings that can aware neuroradiologists in emergency room setting. Abdominal plain radiography is a useful tool for diagnosing and predicting death and complications in these patients considering radiopacity of this pesticide (19, 20). Nikooghadam et al. (21), accidentally discovered similar radiopaque material in the stomach of a patient presenting with abdominal pain and suspected to have aortic dissection.

A limitation in this case was the lack of MR-imaging. The patient could not be sent for an MR because of his unstable vital signs. Furthermore, we were unable to evaluate and measure mitochondrial disorder biomarkers. Many AlP-poisoned patients die before any neurological evaluations mainly due to their unstable vitals making it difficult to move them for imaging studies. Further studies may reveal similar abnormalities in patients with acute ALP poisoning.

## Data availability statement

The original contributions presented in the study are included in the article/supplementary material, further inquiries can be directed to the corresponding author/s.

## Ethics statement

Ethical review and approval was not required for the study on human participants in accordance with the local legislation and institutional requirements. Written informed consent from the patients/participants or patients/participants' legal guardian/next of kin was not required to participate in this study in accordance with the national legislation and the institutional requirements. Written informed consent was obtained from the patient for the publication of any potentially identifiable images or data included in this article.

## Author contributions

HH-M and NZ treated the patient. MS consulted the patient. SA interpreted the CT findings. SS wrote the first manuscript. All authors were involved in the analysis and interpretation of findings. All authors proofed the manuscript and contributed to important intellectual content. All authors contributed to writing and approved the final manuscript.

## Conflict of interest

The authors declare that the research was conducted in the absence of any commercial or financial relationships that could be construed as a potential conflict of interest.

## Publisher's note

All claims expressed in this article are solely those of the authors and do not necessarily represent those of their affiliated organizations, or those of the publisher, the editors and the reviewers. Any product that may be evaluated in this article, or claim that may be made by its manufacturer, is not guaranteed or endorsed by the publisher.

## Abbreviations

AF: Atrial fibrillation
ARDS: Acute respiratory distress syndrome
ATP: adenosine triphosphate
ALP: Aluminum Phosphide
BE: Base Excess
BP: Blood Pressure
Cr: creatinine
CT: Computerized tomography
EEG: Electroencephalogram
GCS: Glasgow coma scale
GFR: Glomerular Filtration Rate
HIE: Hypoxic-ischemic encephalopathy
ICU: Intensive Care Unit
MR: Magnetic resonance
MDD: Major Depressive Disorder
PR: Pulse Rate
RR: Respiratory Rate
VBG: Venous Blood Gas.
